# Clinicopathologic significance of claudin-6, occludin, and matrix metalloproteinases −2 expression in ovarian carcinoma

**DOI:** 10.1186/1746-1596-8-190

**Published:** 2013-11-19

**Authors:** Liping Wang, Xiangshu Jin, Dongjing Lin, Zhijing Liu, Xiaowei Zhang, Yan Lu, Yuanyuan Liu, Min Wang, Minlan Yang, Jiuxia Li, Chengshi Quan

**Affiliations:** 1 The Key Laboratory of Pathobiology, Ministry of Education, College of Basic Medical Sciences, Jilin University, Changchun, Jilin, China

**Keywords:** Tight junctions, Ovarian cancer, Claudin-6, Occludin, MMP-2

## Abstract

**Background:**

Tight junctions (TJs) are mainly composed of claudins, occludin, and tight junction adhesion molecules (JAM). The invasive and metastatic phenotype of highly invasive cancer cells has been related to abnormal structure and function of TJs, and with expression of activated matrix metalloproteinases (MMPs). The relevance of these mechanisms responsible for the invasion and metastasis of ovarian carcinoma is unclear. Similarly, it is not known if the expression of claudin-6, occludin and MMP2 is related with the clinical properties of these tumors.

**Methods:**

Expression of claudin-6, occludin, and MMP2 was detected in samples of human ovarian cancer tissues by immunohistochemistry and correlated with the clinical properties of the tumors.

**Results:**

The positive expression rates of claudin-6 and MMP-2 were higher in ovarian papillary serous carcinomas than n ovarian serous adenomas (*P* < 0.05). There were no differences in the expression of occludin (*P* > 0.05). The expression of claudin-6 and occludin in ovarian cancer was not correlated with patient age, pathological grade, clinical stage, and metastasis (*P* > 0.05). MMP-2 expression was enhanced with increased clinical stage and metastasis (*P* < 0.05), but was unrelated to patient age or tumor grade (*P* > 0.05). There were no apparent correlations between expression of claudin-6, occludin and MMP-2 in ovarian cancer tissue (*P* > 0.05).

**Conclusions:**

Our data suggest, for the first time, that the claudin-6 and MMP-2 are up-regulated in ovarian papillary serous carcinomas, MMP-2 expression was enhanced with increased clinical stage and metastasis. Claudin-6 and MMP-2 may play a positive role in the invasion and metastasis of ovarian cancer.

**Virtual slides:**

The virtual slide(s) for this article can be found here: http://www.diagnosticpathology.diagnomx.eu/vs/1775628454106511.

## Introduction

Ovarian cancer is one of the most common neoplasms in women and epithelial tumors account for approximately 90% of ovarian malignancies [1]. The onset of ovarian cancer is often asymptomatic and not easily detected, but nevertheless often associated with early metastasis and a poor prognosis [2].

The tight junctions (TJs) are mainly located in the junctional complex of epithelial and endothelial cells, and comprise three essential membrane proteins: claudins, occludins, and tight junction adhesion molecule (JAM) [3]. There are 27 members in claudins family, they have variable tissue-specific expression manners [4]. The aberrant expression of the claudin proteins has been related to various human carcinomas. Some studies have reported that down-regulation of the claudin, occludin and other TJ proteins has been considered one of the important reasons for the loss of cell adhesion, cell polarity, invasion and metastasis of malignant tumors [3], up-regulation of some TJ proteins has also been associated with the malignant phenotype. But how increased claudins expression contributes to tumor progression is less clear. On the one hand, claudins up-regulation is related to a significant disorganization of the tight junction strands and increased paracellular permeability [5-9], which have been related to the invasive and metastatic phenotype of highly invasive cancer cells [10]. On the other hand, claudins have been shown to promote tumor invasion by the activition of MMPs [5,11,12].

MMPs are a zinc ion endopeptidase enzyme family, and the main enzymes responsible for degradation of extracellular matrix and cancer invasion and metastasis [13,14]. Their substrates are type IV collagen and the laminin layer in the basement membrane [15].

This research was conducted to examine the expression of claudin-6, occludin and MMP-2 in 36 ovarian papillary serous carcinomas and 26 ovarian serous adenomas specimens, and investigate the relationship between their expression and clinical properties of the ovarian tumor patients.

## Materials and methods

### Patients and tissue specimens

A total of 62 ovarian cancer patients received surgery in the first hospital of Jilin University between January 2006 and October 2007. A total of 36 ovarian papillary serous carcinoma specimens from patients ranging in age from 14 to 80 years (mean age 47 years) were included in this study. Pathological examination confirmed the presence of tumor metastasis in 13 cases but these were not identified in 23 cases. The disease stages of all the patients were classified according to TNM clinical stage (UICC 1997 staging criteria). There were 20 cases of Stage I and II ovarian cancer, 16 cases of Stage III and Stage IV cancer. Fourteen of the carcinomas were of pathological grade I, 12 cases grade II, and 10 cases of grade III. None of the cancer patients had received anticancer therapy preoperatively. Twenty-six ovarian serous adenomas served as experimental controls. The clinicopathological features of the patients are summarized in Table 1. All specimens were fixed in 10% neutral formalin at room temperature, dehydrated, and embedded in paraffin. For the use of these clinical materials for research purposes, prior patient’s consent and approval from the Ethics Committee of Jilin University was obtained.

**Table 1:** Expression of claudin-6, occludin and MMP-2and clinicopathological characteristics in breast carcinoma patients

**Variables**	**No.**	**Claudin-6**			**Occludin**			**MMP-2**		
		**Positive**	**Negative**	***p***	**Positive**	**Negative**	***p***	**Positive**	**Negative**	***p***
**Patient Age**										
20-40	9	6	3	0.696^*^	4	5	0.846^*^	6	3	0.900^*^
41-60	20	14	6		11	9		15	5	
61-80	7	5	2		4	3		5	2	
**Histology**										
Ovarian papillary serous carcinoma	36	25	11	0.007	19	17	0.108^*^	26	10	0.001
Ovarian serous adenomas	26	9	17		19	7		8	18	
**Pathological Grade**										
I	14	9	5	0.696^*^	8	6	0.918^*^	9	5	0.6820^*^
II	12	8	4		6	6		9	3	
III	10	8	2		5	5		8	2	
**Clinical Stage**										
I-II	20	13	7	0.777^*^	10	10	0.709^*^	11	9	0.028
III-IV	16	12	4		9	7		15	1	
**Metastasis**										
Positive	13	11	2	0.267^*^	7	6	0.923^*^	12	1	0.037
Negative	23	14	9		12	11		14	9	

chi-square test.

*No statistical significance.

### Immunohistochemistry staining

Four-micrometer-thick tissue sections were cut from the paraffin-embedded blocks. Deparaffinization was performed using a solution containing xylene, and the sections were rehydrated with graded ethanol. The slides were placed in target retrieval solution (citrate buffer, pH 6.0) and boiled for 5 min in a microwave oven. After the samples had cooled for 30 min, endogenous peroxidase activity was inhibited by treatment with 3% H_2_O_2_ for 30 min. The sections were washed with PBS three times. After a 30-min protein block with normal goat serum or normal rabbit serum, the samples were incubated with the following antibodies overnight at 4°C: anti-claudin-6 (1:100; Santa Cruz Biotechnologies, Santa Cruz, CA, USA), anti-occludin (1:100; Santa Cruz Biotechnologies, Santa Cruz, CA, USA), anti- MMP-2 (1: 100; Santa Cruz Biotechnologies, Santa Cruz, CA, USA). Immunostaining was performed using the streptavidin-biotin-peroxidase complex. The biotin-conjugated secondary antibody was incubated for 20 min at room temperature. The colour reagent diaminobenzidine (Bios, Beijing, China) was used to visualize the bound antibody. The sections were counterstained with Mayer’s haematoxylin. The negative controls were handled in the same way but were incubated in PBS instead of primary antibody.

Negative controls were processed as above without the primary antibody or normal IgG. Ovarian cancer tissues sections with positive claudin-6, occludin and MMP-2 were stained to serve as pisitive controls. The brown staining of claudin-6, occludin and MMP-2 on the cell membrane and/or cytoplasm were as classified as positive staining. Immunostaining was observed under light microscopy with 400× magnification, and positive cells, negative cells and total cells of five different visual fields were numbered in each specimen. Scoring was performed as follows: negative (−), <5% positive tumor cells; positive (+), ≥5% positive tumor cells.

### Statistical analyses

All statistical analysis were performed using SPSS 12.0 software. Chi-square test and McNeamr test methods were used respectively according to the type of data to analyze whether there were significant differences or relevance between the various indicators. *P* < 0.05 was considered statistically significant.

## Results

### Claudin-6 expression in ovarian cancer tissues and its clinical significance

**Figure 1:**
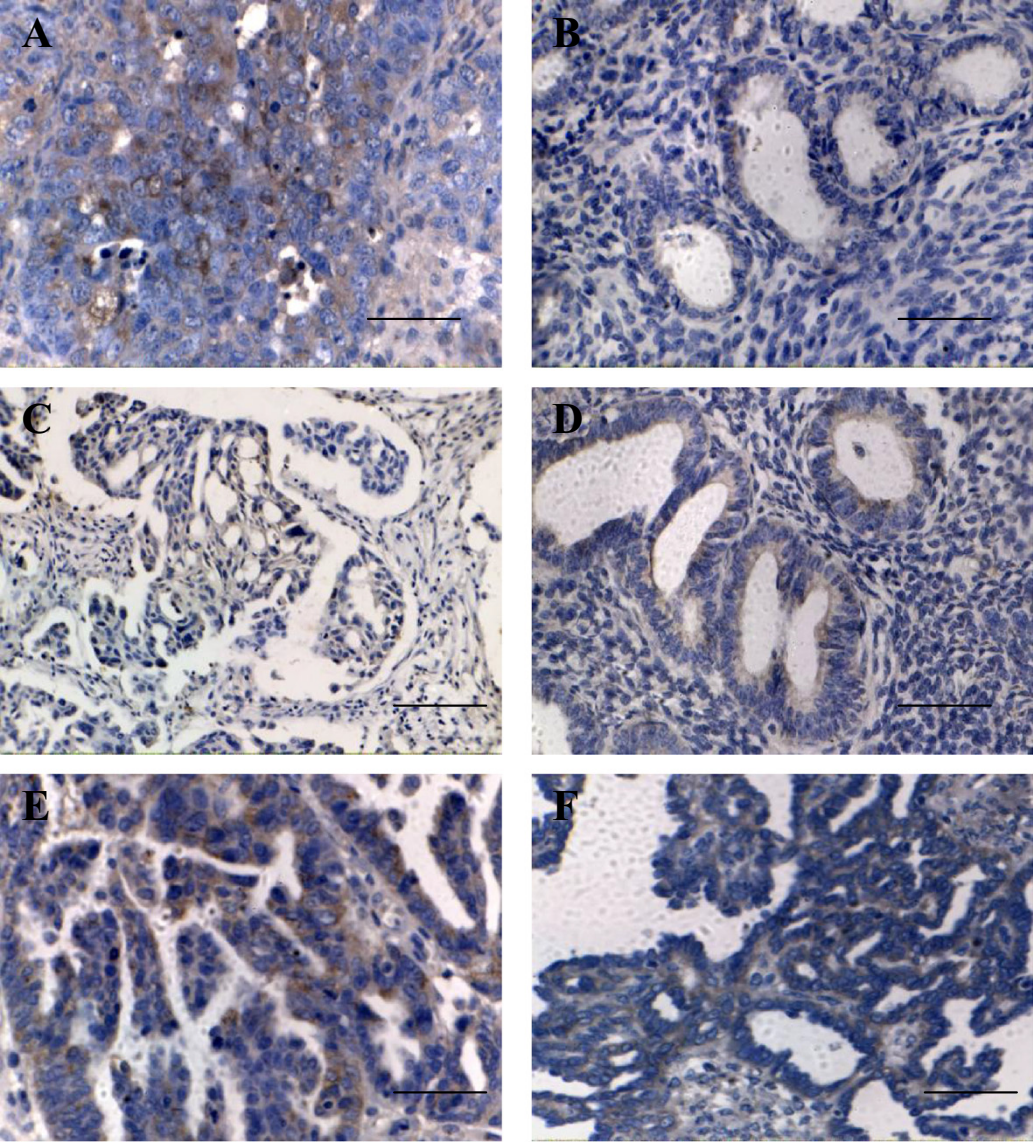
**The immunohistochemical expression of claudin-6, occludin and MMP-2 in ovarian papillary serous carcinoma and ovarian serous adenoma. A**, Claudin-6 was strongly expressed in ovarian carcinoma; **B**, Claudin-6 was weakly expressed in ovarian serous adenoma; **C**, Occludin expression in ovarian carcinoma; **D**, Occludin expression in ovarian serous adenoma; **E**, MMP-2 was strongly expressed in ovarian carcinoma; **F**, MMP-2 was weakly expressed in ovarian serous adenoma. [bar line = 50 μm].

Immunohistochenical staining results revealed that claudin-6 protein was localized in both the cell membrane and cytoplasm. Expression of claudin-6 was 69.4℅ (25/36) in the ovarian carcinomas and 34.6℅ (9/26) in the ovarian serous adenomas and expression of claudin-6 in ovarian papillary serous carcinomas significantly was significantly higher than that in ovarian serous adenomas (Table 1, Figure 1A and 1B, chi-square test χ^2^ = 7.3946, *P =* 0.0065). As shown in Table 1, the expression of claudin-6 was not associated with pathological grade (*P* = 0.6964), clinical stage (*P* = 0.7771), metastasis (*P* = 0.2674) or patient age (*P* = 0. 6964) in this study.

### Occludin expression

Immunohistochemical staining indicated that occludin was located in the membrane or cytoplasm (Figure 1C and 1D). The expression of occludin was found in 52. 8% (19/36) of ovarian papillary serous carcinomas and 73.1% (19/26) of ovarian serous adenomas. This difference was not significant (chi-square test χ^2^ = 2.6220,*P* = 0.1082), as shown in Table 1. The expression of occludin was not correlated with pathological grade (*P* = 0.9184), clinical stage (*P* = 0.7090), metastasis (*P* = 0.9231) or patient age (*P* = 0. 8460) (Table 1).

### MMP-2 expression

**Figure 2:**
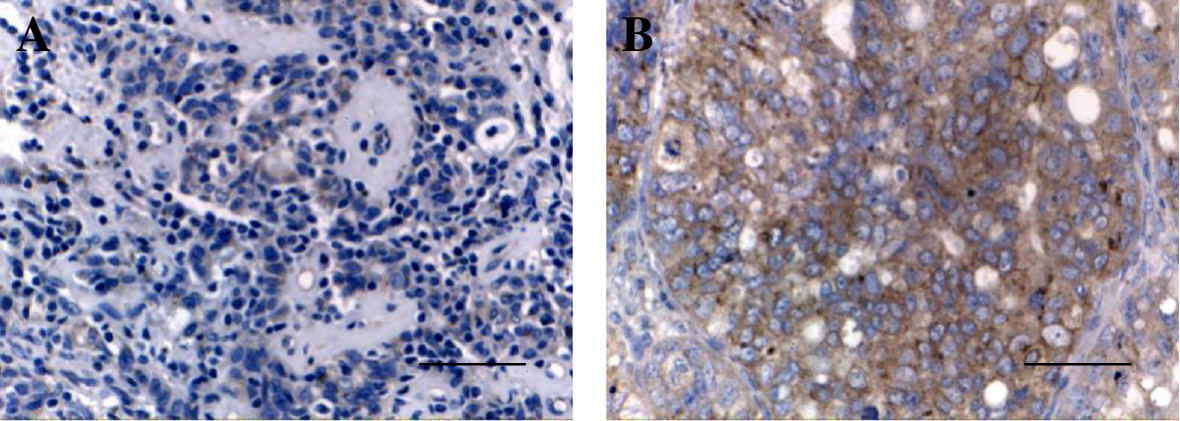
**The expression of MMP-2 in clinical stage I and clinical stage III ovarian carcinoma. A**, MMP-2 was weakly expressed in Clinical stage I ovarian carcinoma; **B**, MMP-2 was strongly expressed in clinical stage III ovarian carcinoma. [bar line = 50 μm].

By immunohistochemistry, MMP-2 expression was located in the membrane or cytoplasm, (Figure 1E, 1F). The expression of MMP-2 was positive in 72.2℅ (26/36) of ovarian papillary serous carcinomas and 30.8℅ (8/26) of ovarian serous adenomas (Table 1). The MMP-2 Protein in ovarian papillary serous carcinomas was significantly elevated relative to ovarian serous adenomas. (chi-square test χ^2^ = 10.4747, *P =* 0.0012 < 0.05). The expression of MMP-2 was positively correlated with clinical stage (*P* = 0.0275) (Table 1, Figure 2A and 2B) and metastasis (*P* = 0.0367) (Table 1). The expression of MMP-2 was not correlated with pathological grade (*P* = 0.6820) or patient age (*P* = 0. 8996) (Table 1).

### Correlation of claudin-6, occludin and MMP-2 expression in ovarian carcinoma

Correlation between the expression of claudin-6, occludin and MMP-2 was evaluated in ovarian carcinoma specimens by McNemar test. The expression of claudin-6 was not correlated with that of occludin (McNemar test χ^2^ = 2.5714*, P =* 0.1088 *>* 0.05) or MMP-2 (McNemar test χ^2^ = 1.0000*, P =* 0.3173 *>* 0.05) (Table 2). Similarly, the expression of occludin was not correlated with MMP-2 ( McNemar test χ^2^ = 2.0000*,P =* 0.1573) (Table 3).

**Table 2:** Correlation between the expression of MMP-2, occludin and claudin-6

**Item**	**Occludin (+)**	**Occludin (−)**	***P***	**MMP-2(+)**	**MMP-2(−)**	***P***
Claudin-6(+)	14	10	0.109^*^	17	10	0.317^*^
Claudin-6(−)	4	8		6	3	

McNemar test.

*No statistical significance.

**Table 3:** Correlation between the expression of MMP-2 and occludin

**Item**	**Occludin (+)**	**Occludin (−)**	***P***
MMP-2(+)	10	6	0.157^*^
MMP-2(−)	12	8	

McNemar test.

*No statistical significance.

## Discussion

TJs located in the junctional complex of epithelial cells and endothelial cells, consist of three essential membrane proteins: claudins, occludins and JAM. These maintain cell polarity, adhesion, and regulate cell proliferation and differentiation [16]. Recent studies suggest that the invasive and metastatic characteristics of ovarian cancer may be associated with abnormal structure and function of TJs in addition to activation of matrix metalloproteinases [5]. Currently the possibility that abnormalities in TJ s protein expression relate to the invasion and metastasis of ovarian cancer has not been widely examined. Similarly, studies concerning the correlation of claudin-6, occludin and MMP-2 with ovarian cancer have rarely been reported. In this study, we used immunohistochemistry to detect the expression of claudin-6, occludin and MMP-2 in 36 cases of ovarian papillary serous carcinomas and 26 patients with ovarian serous adenomas. We looked for correlations between the expression of these parameters and clinical stage, histological grade and metastasis. Our data show that the positive expression rates of claudin-6 and MMP-2 were higher in ovarian papillary serous carcinoma than in ovarian serous adenomas specimens. Also, MMP-2 expression was enhanced with increased clinical stage, and metastasis. We speculate that the up-regulation of claudin-6 plays a role in ovarian cancer carcinogenesis and metastasis by influencing structure and function of TJs or promoting the activation of MMP-2 that degrades the extracellular matrix, and can be used as an index for the prognostic assessment of ovarian cancer carcinogenesis and metastasis.

### Expression of Claudin-6 in ovarian cancer tissue and its significance

The loss of cell polarity and abnormal permeability is a characteristic change in malignant epithelial tumors [17,18]. TJs are the key structures that maintain these functions in epithelial cells, but structure and function of TJs are changed in many epithelial tumors. Claudins aberrant regulation was related to abnormal structure and function of TJs [9]. It has been reported that several kinds of tumors have relation with up-regulation of claudins. For example, claudin-3 and claudin-4 were expressed at high levels in endometrioid adenocarcinoma [19] and prostate cancer [20]; claudin-10 expression was up-regulated in hepatocellular carcinoma [21] and thyroid papilloma [22]; claudin-2 has been reported to be up-regulated in thyroid cancer and endometrial cancer [23]. Claudins have also been shown to promote tumor invasion by the activation of MMPs [5,11,12]. Similarly, down-regulation of several claudin proteins has also been reported to promote tumor invasion by the reduction of TJs function in various cancers. For example, down-regulation of claudin-1 has been found in colon cancer [24]. Down-regulation of claudin-7 has been found in invasive breast cancer [25]. These reports of down-regulated TJs expression in cancer are consistent with the generally accepted idea that tumorigenesis is accompanied by a disruption of TJs, a process that may play a critical role in the loss of cohesion and invasiveness observed in cancer cells. Claudin-6 belongs to the family of claudin proteins. We found previously that, claudin-6 was down-regulated in breast cancer cells [26,27] as well as in gastric cancer [28], but up-regulated in ovarian cancer cells. We recently found claudin-6 inhibited invasion in breast cancer cells by reversing EMT (data not show). Together, the up-regulation and down-regulation of the claudin proteins are both related to various human cancers due to high tissue-specific expression manners of claudin proteins. We speculated that aberrant expression of claudin-6 protein may play a key role in the invasion and metastasis of ovarian cancer and other cancers.

Our data show that the expression of claudin-6 in 36 ovarian papillary serous carcinomas is significantly higher than in 26 ovarian serous adenomas; claudin-6 expression in metastatic tumors was significantly higher than in the tumors without metastasis. Though this difference was not statistically significant, it suggests that up-regulation of caudin-6 may play a role in the occurrence, invasion and metastasis of ovarian cancer. It is unclear why there is elevated expression of claudins and associated destruction of TJs in tumorigenesis and metastasis. However, it seems possible that the aberrant up-regulation of claudins may alter the constituent ratio of TJs. On the one hand, it may cause changes in the structure of TJs leading to loss of the integrity of TJ function, increases in cell gap and loss of robustness of the cell junction. On the other hand, it may lead to disappearance of cell polarity, weakening of intercellular adhesion and facilitation of invasion. These changes may regulate tumor cell proliferation, differentiation, survival and apoptosis though some signal transduction pathways, which play a role in occurrence, invasion and metastasis of tumor. In addition, up-regulation of claudins may activate MMPs that also promote invasion and metastasis of tumors. We speculate that up-regulation of claudin-6 may have the same mechanism play a role in occurrence, invasion and metastasis of ovarian cancer.

### Occludin expression in ovarian cancer tissue and its significance

Occludin is a transmembrane protein that has an important role in maintaining function of TJs [29]. Our results show that occludin expression in is low in comparison with its expression in ovarian serous adenomas, occludin expression is less in poorly differentiated ovarian carcinoma than in well-differentiated tumors. Although this result was not statistically significant, it suggests that occludin expression in ovarian cancer may play a role in the occurrence and development of ovarian tumors. Currently relevant information found occludin expression to be low in a variety of tumors, such as endometrial cancer tissue and in human prostate cancer tissue [30-32]. Osanai et al. [33] transfected the occludin gene into tumor cells and found that it can affect the phenotype of the tumor cells, in particular by increasing the sensitivity of transfected tumor cells to apoptotic factors, thereby acting as a tumor suppressor. It has been suggested that inhibition of occludin may act on TJs directly or indirectly to bind with factors that regulate cell proliferation, differentiation and cell cycle, receiving and imparting PKC and Rho protein etc. molecular signaling, regulating tumor cell survival [34].

### MMP-2 expression in ovarian cancer tissue and its significance

Campo et al. [35] found MMP-2 was undetectable or expressed at low levels in benign ovarian tumors and low-grade malignant tumors without invasion. Such tumors had a continuous basement membrane. In contrast, MMP-2 expression was high and basement membranes were widely missing in aggressive tumors accompanied by distant organ and lymph node metastasis. MMP-2 and MMP-9 have been related to poor prognosis and lung metastasis in breast cancer [36,37]. Subsequently Autio-Harnainen [38] found expression of MMP-2 in both epithelial and stromal cells by immunohistochemistry in their studies of ovarian tumors. Our studies showed that MMP-2 expression was higher in ovarian papillary serous carcinomas than in ovarian serous adenomas. MMP-2 expression in ovarian cancer was higher as the clinical stage increased and MMP-2 positive expression was significantly higher (P < 0.05) in ovarian cancers with metastasis than without metastasis. This result suggests that high expression of MMP-2 may be related to the invasion and metastasis of ovarian cancer and supports the view of Parsons [39], that high expression of MMP-2 in ovarian cancer suggests that invasion and metastasis has occurred.

### Correlation of expression of claudin-6, occluding and MMP-2

Our correlation studies showed that expression of the TJ proteins claudin-6, occludin and MMP-2 were not correlated in ovarian cancer. The experimental results show that the expression of MMP-2 was up-regulated in ovarian cancer. Claudin-6 was also increased. To some extent, the high expression of MMP-2 may be due to increased Claudin-6 expression, thus speeding up the extracellular matrix degradation and resulting in the damage to the structure of TJs. Agarwal et al. [5] found that up-regulation of claudin-3 and claudin-4 in ovarian cancer promoted metastasis of ovarian cancer. These workers thought that the up-regulation of claudin-3 and claudin-4 may take effect by increasing MMP-2. MMP-2 not only regulates the adhesion between cells thereby affecting invasive metastasis of tumor cell, but also induce activation of extracellular protein, and plays a key role in attracting inflammatory cells and spontaneously stimulating migration of tumor cells [40]. MMP-2 can also promote tumor angiogenesis by rebuilding the extracellular matrix [41]. MMP2 is a prognostic indicator in patients with lymph node-negative breast carcinomas [42]. Many genes can be significant prognostic and diagnostic markers for ovarian tumors. For example, estrogen maybe a diagnostic marker in some kinds of tumor [43], Eag is a prognostic indicator in ovarian cancer [44], p53 and metallothionein may be helpful in the typing of borderline and malignant ovarian tumors [45]. The difference in expression of P53, MAPK, topoisomerase II alpha and Ki67 between low and high-grade group indicate these genes may be the prognostic markers in ovarian cancer [46]. To determine whether MMP2 is a prognostic indicator in ovarian tumors need further researches in the future.

Our present study shows that claudin-6 and MMP-2 were both up-regulated in ovarian cancer, MMP-2 expression was enhanced with increased clinical stage and metastasis indicates that claudin-6 and MMP-2 may play an important role in the progression of ovarian cancer. However, the correlations between claudin-6 expression with occludin and MMP-2 expression have not been observed.

## Conclusion

In summary, the up-regulation of claudin-6 may result in abnormal structure and function of TJs and activation of MMP-2 both of which can result in some of the highly invasive tumor cells acquiring invasive and metastatic phenotype. Claudin-6 and MMP-2 can be used as important indicators for the judgment of malignant behavior of ovarian cancer such as invasion and metastasis. However, the exact mechanism responsible for the occurrence, progression and metastasis of ovarian cancer is not completely understood and needs further studies.

## Competing interests

The authors declare that they have no competing interests.

## Authors’ contributions

CQ carried out part of the experiments, participated in the design of the study, performed the statistical analysis, and drafted the manuscript. LW, XJ, and DL carried out most of experiments, and helped draft the manuscript. ZL, XZ, YL, YL, MW, MY, and JL assisted with the experiments, and helped to edit the paper. All authors have read and approved the final manuscript.
